# Does Cognitive Control Help or Hurt Social Anxiety? Conditional Effects of Response Inhibition on Safety Behavior Use and Post-event Processing

**DOI:** 10.1007/s10608-026-10746-x

**Published:** 2026-04-24

**Authors:** Alexandra Marie Adamis, John Sanford Gardner, Bunmi Olatunji

**Affiliations:** Vanderbilt University, Nashville, United States

**Keywords:** Social anxiety, Cognitive control, Inhibition, Safety behaviors, Post-event processing

## Abstract

**Purpose:**

Although safety behaviors (SBs) and post-event processing (PEP) play an important role in the maintenance of social anxiety, less is known about the individual differences that might influence one’s use of these maladaptive cognitive-behavioral regulatory strategies. This study examined whether response inhibition, a form of cognitive control involving one’s ability to override automatic impulses, moderated the relationship between social anxiety and engagement in SBs and PEP.

**Methods:**

Adults with high and low levels of social anxiety completed a stop-signal task (SST) measuring response inhibition abilities, then completed self-report assessments of past-week SB use (*n* = 192) and PEP (*n* = 183) following an impromptu laboratory speech task one week later.

**Results:**

Moderation analyses revealed that social anxiety levels interacted with response inhibition abilities to predict both SBs and PEP, but in unexpected directions. While higher response inhibition abilities predicted lower levels of SBs and PEP for those with low social anxiety, this pattern was reversed for those with high social anxiety, for whom better response inhibition was associated with *higher* levels of SBs and PEP.

**Conclusions:**

Findings indicate that response inhibition is not universally protective; rather, its effects might depend on symptom severity and individuals’ (mal)adaptive goals in social contexts. The implications of these findings for existing cognitive-behavioral models of social anxiety disorder are discussed.

**Supplementary Information:**

The online version contains supplementary material available at https://doi.org/10.1007/s10608-026-10746-x.

## Introduction

Social anxiety disorder (SAD) is one of the most common psychiatric disorders, characterized by excessive fear and avoidance of negative evaluation in social situations (American Psychiatric Association, 2022). In the absence of treatment, individuals with SAD often experience chronic symptoms and significant social and occupational impairment (M. B. Stein & Kean, 2000). Although evidence-based treatments for SAD such as cognitive-behavioral therapy (CBT) are often effective (Bandelow et al., 2015), non-response and relapse rates remain substantial (Lorimer et al., 2021; Springer et al., 2018). Therefore, it is crucial to obtain a better understanding of the mechanisms that maintain social anxiety to improve the efficacy of existing interventions. Cognitive-behavioral models of SAD suggest that social anxiety is maintained through a self-reinforcing cycle in which fears of negative evaluation elicit subtle avoidance and ruminative behaviors that inadvertently strengthen perceptions of social danger (Clark & Wells, 1995; Hofmann, 2007). Although safety behaviors (SBs) and post-event processing (PEP) are identified as two central processes that maintain social anxiety in such models, the mechanism(s) that might influence these processes are unclear.

SBs are subtle avoidance or impression management strategies intended to reduce the likelihood of one’s feared outcome (e.g., negative social evaluation; Cuming et al., 2009). For example, individuals with SAD might rehearse speech content, avoid eye contact, or closely monitor their body language to minimize perceived social risks (Clark & Wells, 1995). Although such behaviors might reduce one’s acute distress in the short-term, SBs function to maintain social anxiety in the long-term by preventing corrective learning and/or making feared outcomes more likely (Clark & Wells, 1995; Hofmann, 2007). Indeed, SBs commonly reinforce the maladaptive beliefs underlying SAD via “misattributions of safety,” meaning individuals mistakenly conclude that their feared outcome (e.g., social rejection) did not occur due to their use of SBs, rather than due to the relatively low likelihood and/or severity of their fear (Salkovskis, 1991). From an inhibitory learning perspective, SBs might further impede the potential benefit of exposure to feared social situations by reducing expectancy violations and reinforcing distress intolerance (Blakey & Abramowitz, 2016). Additionally, SBs could paradoxically increase the likelihood of the very feared outcomes they are intended to prevent (Clark & Wells, 1995). For example, Rowa and colleagues (2015) found that SB use predicted poorer observer-rated performance on a speech task amongst a sample of patients with SAD, demonstrating that SBs undermine objective social performance. Considered alongside experimental work demonstrating that SB reduction alone has therapeutic effects on social anxiety severity (Cougle et al., 2020; Zech et al., 2025), it is clear that SBs play an important causal role in the maintenance of social anxiety.

Following social interactions, socially anxious individuals commonly engage in PEP, a form of repetitive negative thinking about past social experiences (Flynn & Yoon, 2025). Akin to a “postmortem” analysis, PEP involves conducting a detailed mental review of a past social interaction, with a particular focus on one’s own performance and the ambiguous social feedback one received (Flynn & Yoon, 2025). Unfortunately, because socially anxious individuals tend to allocate disproportionate attention to negative internal stimuli (e.g., anxious thoughts and feelings; Adamis et al., 2025b) and signals of negative evaluation (Günther et al., 2021) during social interactions, PEP is often negatively biased and accordingly is theorized to reinforce maladaptive beliefs about one’s social inadequacy (Clark & Wells, 1995; Flynn & Yoon, 2025; Hofmann, 2007). Consistent with this view, Zhang et al. (2025) recently found that PEP mediated the relationship between self-focused attention and social anxiety severity, suggesting that PEP might explain why maladaptive patterns of information processing during social events result in the longer-term maintenance of social fears. Laboratory work complements these findings, demonstrating that experimentally induced PEP engenders increased anxiety surrounding social stressors (Rowa et al., 2014; Wong & Moulds, 2009). Furthermore, reductions in PEP have been shown to correlate with reductions in social anxiety symptoms in the context of cognitive-behavioral treatments for SAD (Abbott & Rapee, 2004; McEvoy et al., 2009; Price & Anderson, 2011), suggesting that PEP might be an important target that mediates treatment gains.

Taken together, prior empirical and theoretical work converges to suggest that SBs and PEP are core mechanisms involved in the maintenance of social anxiety. Thus, it is unsurprising that CBT for SAD commonly aims to reduce SBs and PEP throughout the course of treatment (e.g., Stangier et al., 2003). However, limitations to the efficacy of CBT for SAD underscore the need for further investigation into factors that influence the extent to which socially anxious individuals enact, and accordingly might have difficulty reducing reliance on, these maladaptive behaviors (Lorimer et al., 2021; Springer et al., 2018). Top-down regulatory capabilities such as cognitive control might constitute an important determinant of the extent to which socially anxious individuals revert to maladaptive behaviors in the context of social threats. Cognitive control encompasses a set of top-down, “executive” functions (e.g., inhibition, set-shifting, working memory updating) that underlie one’s ability to regulate cognitive and behavioral responses in a goal-directed manner (Miyake et al., 2000). Strong cognitive control abilities are largely conceptualized as a protective factor that underlies the complex reasoning, adaptation, problem-solving, and emotion regulation required for adaptive functioning (Zelazo, 2020).

Cognitive control *deficits* are considered a transdiagnostic risk factor for psychopathology broadly (Zelazo, 2020), and anxiety disorders specifically (Snyder et al., 2015), in part because they might impair one’s ability to override habitual threat-driven or fearful responses in favor of adaptive, goal-directed behavior. Within the context of social anxiety in particular, individual differences in cognitive control might constitute an important determinant of how individuals either adaptively regulate or exacerbate their anxious responses to social threats. For instance, cognitive control abilities might influence whether one is able to override habitual avoidance tendencies during social situations and/or inhibit a perseverative focus on social failures after social events. Consistent with this view, prior research demonstrates that lower cognitive control abilities are associated with greater attentional processing of social threats (Taylor et al., 2016), increased enactment of SBs in social contexts (Adamis et al., 2025a), and higher levels of trait PEP (Sluis et al., 2018). However, much of this prior work assesses cognitive control functions via self-report measures, and/or examines associations between cognitive control and anxious patterns of behavior exclusively within highly socially anxious samples. Thus, it remains unclear if poor cognitive control is also associated with social anxiety maintenance mechanisms when *behaviorally* assessed, as well as whether cognitive control functions differently for those that are socially anxious relative to those who are not.

To address these gaps in the literature, the present study investigated whether individual differences in cognitive control moderated the associations between social anxiety and the use of SBs and PEP surrounding social situations. In examining these interactions, the current research aimed to clarify whether cognitive control functions to attenuate maladaptive maintenance patterns in socially anxious individuals, and accordingly might constitute a promising adjunct treatment target. As a subtype of cognitive control that is particularly relevant for behavioral and cognitive self-regulation, we behaviorally assessed individual differences in response inhibition, or one’s ability to suppress or override prepotent/automatic responses (Bari & Robbins, 2013; Verbruggen & Logan, 2008). Given that response inhibition abilities might help socially anxious individuals override habitual threat-driven responding to instead enact more adaptive social behaviors (Adamis et al., 2025a; Sluis et al., 2018; Taylor et al., 2016), we predicted that stronger response inhibition would be associated with lower utilization of SBs and PEP, particularly amongst individuals high in social anxiety.

## Methods

### Participants

The enrolled sample included 202 adults (70.80% female; *M*_age_ = 26.84, *SD* = 13.86) recruited from a southeastern university and surrounding areas (see Table 1). Participants aged 18 or older were eligible for the study on the basis of cutoff scores on the Social Interaction Anxiety Scale–6 (SIAS-6): those scoring ≥ 7 were classified as “high social anxiety” and those scoring ≤ 2 were classified as “low social anxiety” (Peters et al., 2012). No additional exclusion criteria were applied. On the basis of these eligibility criteria, *n* = 108 participants were enrolled in the high social anxiety group, and *n* = 94 were enrolled in the low social anxiety group. Groups did not significantly differ on race/ethnicity, but did demonstrate differences in age (*t* = 2.25, *p* = 0.03) and gender identity (*X*^*2*^ = 6.31, *p* = 0.04). On average, the high social anxiety group was younger and more likely to select “Other” for their gender identity than the low social anxiety group, as is reflective of typical demographic characteristics of patients with SAD (i.e., younger age, sexual/gender identity minorities; Mahon et al., 2022; D. J. Stein et al., 2017).

**Table 1:** Group differences in demographics and study variables

Variable	Low social anxiety (*n* = *94)*	High social anxiety (*n* = *108)*	*t* or *X*^*2*^	*df*	*p*
Age	29.16 (15.84)	24.81 (11.57)	2.25	200	0.026
*Gender identity*			6.31	2	0.043
Female	69 (73.40)	74 (68.52)			
Male	25 (26.60)	27 (25.00)			
Other	0 (0.00)	7 (6.48)			
*Racial/Ethnic identity*					
White	64 (68.09)	64 (59.26)	1.69	1	0.194
African American/ Black	10 (10.64)	19 (17.59)	1.98	1	0.160
Hispanic/Latino	11 (11.70)	5 (4.63)	3.45	1	0.063
Asian/Pacific Islander	20 (21.28)	24 (22.22)	0.03	1	0.871
Native American	1 (1.06)	0 (.00)	1.16	1	0.283
Other	1 (1.06)	2 (1.85)	0.21	1	0.644
*Participant type*			2.12	1	0.146
Student	57 (60.64)	76 (70.37)			
Community Member	37 (39.36)	32 (29.63)			
*Study variables*					
SSRT	277.80 (79.61)	264.55 (49.56)	1.42	195	0.157
SIAS	17.01 (9.96)	37.81 (12.70)	− 12.81	200	< 0.001
SAFE	22.03 (14.68)	38.86 (17.92)	− 7.15	195	< 0.001
SARQ	13.55 (10.45)	26.12 (11.41)	− 7.83	186	< 0.001

Data are presented as Mean (*SD*) or *n*(%); SSRT: Stop Signal Reaction Time; SIAS: Social Interaction Anxiety Scale; SAFE: Subtle Avoidance Frequency Estimation; SARQ: Socially Anxious Rumination Questionnaire

### Measures

#### Stop-Signal Task (SST; Verbruggen & Logan, 2008)

Response inhibition was assessed using a computerized Stop-Signal Task programmed in E-Prime 3.0. Participants were instructed to respond to go-signals (i.e., shapes) via key presses (e.g., “Press X for triangles,” “Press M for circles”) as quickly and accurately as possible. In approximately 25% of trials, an auditory stop-signal (i.e., a beep) was presented through headphones after a variable stop-signal delay (SSD; i.e., the interval between the onset of the go-signal and the stop-signal). The SSD was dynamically adjusted by ± 50 ms following each stop-signal trial depending on participants’ success inhibiting their responses to maintain approximately 50% successful inhibitions across all trials, ensuring that the task was equally challenging for all participants. Stop-signal reaction times (SSRTs) were the primary outcome of the task, reflecting individual latencies to inhibit prepotent responses. Thus, *higher* SSRT values indicate *poorer* response inhibition abilities.

#### Social Interaction Anxiety Scale (SIAS; Mattick & Clarke, 1998)

The full, 20-item version of the SIAS was administered during Session One to obtain a fuller assessment of participants’ social anxiety severity. The SIAS assesses fear and avoidance of day-to-day social interactions (e.g., “I worry about expressing myself in case I appear awkward”), with items rated from zero (*“Not at all”*) to four (*“Extremely”*). The SIAS demonstrated excellent internal consistency in the present sample (α = 0.94).

#### Subtle Avoidance Frequency Estimation (SAFE; Cuming et al., 2009)

The SAFE was administered during Session Two to assess the frequency with which individuals used SBs in social situations over the past week. Participants rated 32 items describing possible SBs (e.g., “Remain silent”) on a scale from zero (“*Never*”) to four (“*Always”*). Higher total scores indicated greater use of past-week SBs. The SAFE demonstrated excellent internal consistency in the present sample (α = 0.93).

#### Socially Anxious Rumination Questionnaire (SARQ; Donohue et al., 2021)

PEP was measured using the SARQ following a speech task administered in Session Two. Participants rated 12 items assessing how frequently they experienced specific negative, ruminative thoughts (e.g., “I made a lot of mistakes”) in reference to the speech task on a scale from zero *(“Not at all”*) to four (*“Very frequently”*). Higher scores indicated greater engagement in PEP. The SARQ demonstrated excellent internal consistency in the present sample (α = 0.95).

### Procedure

The study was approved by the University Institutional Review Board. This study draws on data from a larger parent project examining patterns of attention associated with social anxiety in daily life (Adamis et al., 2025b), but only procedures relevant to the present analyses are reported here. Student and community participants were recruited through undergraduate courses, community flyers, local research listservs, and ResearchMatch. Potential participants first completed the SIAS-6 as part of an online eligibility screening. Interested and eligible individuals were then contacted and invited to participate.

Participants completed two laboratory sessions. Session One began with informed consent, followed by an administration of the SIAS and SST to assess for baseline levels of social anxiety severity and response inhibition abilities. Session Two occurred one week later. At the beginning of Session Two, participants completed the SAFE to assess for use of SBs in social contexts in the past week. Participants then completed an impromptu speech task, a standardized social-evaluative stressor intended to elicit state social anxiety and serve as an anchor for PEP assessments. In the speech task (modeled after the Trier Social Stress Test; Kirschbaum et al., 1993), participants were asked to deliver a speech explaining why they are qualified for their “ideal job” while they were ostensibly video-recorded and evaluated in-vivo by a research assistant. Participants were told that their speech could last up to ten minutes and ended when participants held up a “stop” sign. While a prop camera was used, speeches were not truly recorded. Research assistants maintained a neutral expression and took notes periodically during participants’ speeches, thus reacting equally ambiguously to all participants.

Approximately one hour after Session Two, participants received a secure link via REDCap to complete the SARQ, referencing the speech they had recently performed. This procedure preserved the intended temporal sequencing of the study: social anxiety levels and cognitive control (response inhibition) were assessed prior to the behavioral (SB use) and cognitive (PEP) outcomes of interest. Undergraduate students received course credit for participation; community participants were compensated with gift cards.

### Data Analytic Plan

Analyses were conducted using Hayes’ (2022) PROCESS macro for SPSS. Two moderation models were tested: one predicting SB frequency (via scores on the SAFE), and the other predicting PEP (via scores on the SARQ). In each model, social anxiety group (0 = low, 1 = high) was the predictor, SSRT (mean-centered) was the moderator, and the interaction term represented the hypothesized moderation. Conditional effects were probed at ± 1 SD of mean SSRT scores. Statistical significance was evaluated at α = 0.05 (two-tailed).

## Results

### Descriptive Statistics

Of the 202 participants who enrolled in the study, SSRT data are missing for five participants (two low social anxiety; three high social anxiety) due to computer malfunctions or excessive environmental noise that compromised task performance. SAFE and SARQ data are missing for five participants (two low social anxiety; three high social anxiety) who did not attend Session Two. An additional nine participants (five low social anxiety; four high social anxiety) who did not respond to the post-Session Two remote questionnaire are missing SARQ data. Listwise deletion was used for participants with incomplete data in moderation models. Thus, the final sample size included in analyses was *n* = 192 for Model 1, and *n* = 183 for Model 2.

As depicted in Table 1, the high social anxiety group demonstrated significantly higher levels of social anxiety symptoms (SIAS: *t* = − 12.81, *p* < 0.001), SB use (SAFE: *t* = − 7.15, *p* < 0.001), and PEP following the laboratory speech task (SARQ: *t* = − 7.83, *p* < 0.001) than the low social anxiety group. Groups did not differ in response inhibition abilities, on average (SSRT: *t* = 1.42, *p* = 0.157).

As depicted in Table 2, social anxiety severity was significantly, moderately correlated with SB use (*r* = 0.63, *p* < 0.001) and PEP (*r* = 0.69, *p* < 0.001) across the full sample. SB use and PEP were also significantly, moderately correlated (*r* = 0.66, *p* < 0.001) across the full sample. This pattern of correlations also manifested when examining the high and low social anxiety groups separately. Across the full sample, response inhibition abilities were not significantly associated with social anxiety symptoms or maintenance factors (*p*s > 0.05). However, in the high social anxiety sample, response inhibition difficulty was mildly inversely associated with PEP (*r* = 0.27, *p* = 0.008), and in the low social anxiety sample, response inhibition difficulties were mildly positively associated with SBs (*r* = 0.25, *p* = 0.018).

**Table 2:** Descriptive statistics and correlations among study variables

	1	2	3	4
Full sample (*n*s = 183–202)				
1. SSRT	–			
2. SIAS	− 0.02	–		
3. SAFE	0.04	0.63**	–	
4. SARQ	− 0.13	0.69**	0.66**	–
Mean	270.74	28.13	31.00	20.30
*SD*	65.49	15.49	18.47	12.62
Range	83.30–868.60	2.00–68.00	0.00–76.00	0.00–47.00
High social anxiety (*n*s = 98–108)				
1. SSRT	–			
2. SIAS	− 0.03	–		
3. SAFE	− 0.09	0.52**	–	
4. SARQ	− 0.27*	0.60**	0.52**	–
Shapiro–Wilk Statistic	0.97*	0.99	0.97*	0.97*
Low social anxiety (*n*s = 85–94)				
1. SSRT	–			
2. SIAS	0.16	–		
3. SAFE	0.25*	0.42**	–	
4. SARQ	0.05	0.48**	0.61**	–
Shapiro–Wilk Statistic	0.59**	0.93**	0.93**	0.91**

SSRT: Stop signal reaction time; SIAS: Social interaction anxiety scale; SAFE: Subtle avoidance frequency estimation; SARQ: Socially anxious rumination questionnaire. *p < 0.05 **p < 0.001

Additionally, Shapiro–Wilk tests were conducted to examine normality of study variables within the high and low social anxiety groups (see Table 2). In the low social anxiety group, results indicated that the distributions of study variables were not normal (*p*s < 0.001) and were positively skewed. In the high social anxiety group, results indicated that the SSRT (*p* = 0.039) and SARQ (*p* = 0.030) were not normally distributed and were negatively skewed, and the SAFE (*p* = 0.033) was not normally distributed and was positively skewed. Importantly, the distributions of *residuals* of Models 1 and 2 were approximately normal (see Supplement for residuals and sensitivity analyses); thus, the assumptions of normality required for moderation analysis were met.

### Moderation of Safety Behavior Use

As depicted in Table 3, the first moderation model tested whether response inhibition abilities (SSRT) moderated the association between social anxiety level (high or low) and SB use (SAFE). The overall model was significant (*F*(3,188) = 18.66, *p* < 0.001) and explained 23% of the variance in SAFE scores. The main effect of social anxiety was significant and positive (*b* = 16.94, *SE* = 2.38, 95% CI [12.25, 21.62], *p* < 0.001), and the main effect of SSRT was also significant and positive (*b* = 0.05, *SE* = 0.02, 95% CI [0.00, 0.09], *p* = 0.038). However, these main effects were qualified by a significant group by SSRT interaction.

**Table 3:** Unstandardized model coefficients for the interaction of social anxiety and response inhibition predicting safety behaviors and post‑event processing

Predictor	Y_1_ safety behaviors (*n* = 192)			Y_2_ post-event processing (*n* = 183)		
	*b*	*SE*	*p*	*b*	*SE*	*p*
SA group	16.94	2.38	< 0.001	12.35	1.60	< 0.001
Response inhibition	0.05	0.02	0.038	0.01	0.01	0.634
SA group*Response inhibition	− 0.08	0.04	0.049	− 0.07	0.03	0.011
Constant	21.78	1.73	< 0.001	13.30	1.17	< 0.001
	R^2^ = 0.23			R^2^ = 0.29		
	*F*(3, 188) = 18.66			*F*(3, 179) = 23.85		

SA group: social anxiety group (0: low social anxiety, 1: high social anxiety); Response Inhibition: Stop Signal Reaction Time (higher values indicate poorer inhibitory control); Safety Behaviors: Subtle Avoidance Frequency Estimation scores; Post-Event Processing: Socially Anxious Rumination Questionnaire scores

**Fig. 1:**
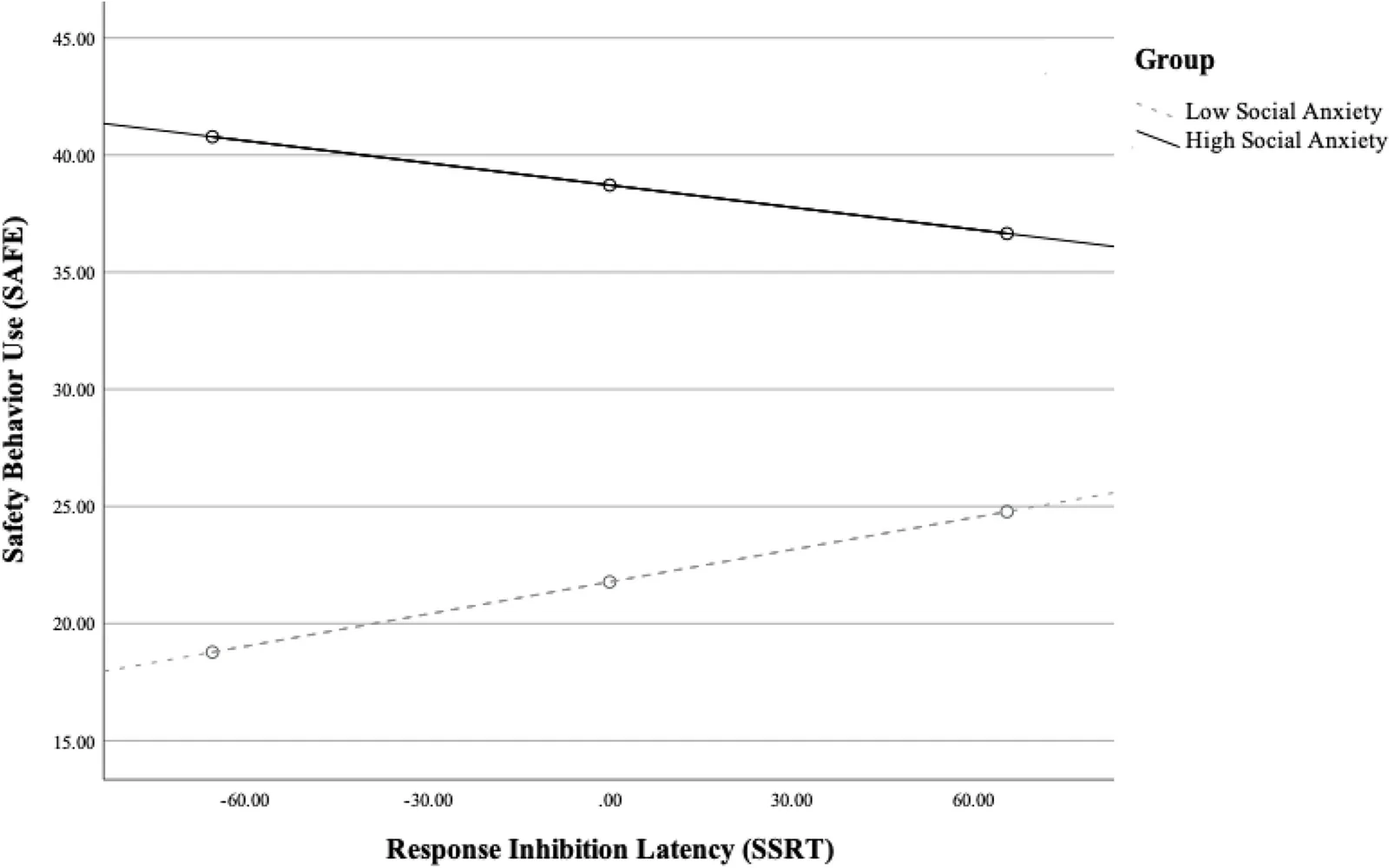
Group differences in safety behavior use (Subtle Avoidance Frequency Estimation [SAFE]) by response inhibition ability (Stop Signal Reaction Time [SSRT]; higher values indicate poorer response inhibition)

Indeed, the interaction between social anxiety and response inhibition was significant (*b* = − 0.08, *SE* = 0.04, 95% CI [− 0.15, − 0.00], *p* = 0.049). Conditional effects indicated that group differences in SB use were largest at lower SSRT values (i.e., better inhibition; − 1 *SD* SSRT: *b* = 22.00, *SE* = 3.44, *t* = 6.39, *p* < 0.001), and smallest at higher SSRT values (i.e., poorer inhibition; + 1 *SD* SSRT: *b* = 11.88, *SE* = 3.55, *t* = 3.35, *p* = 0.001). The interaction between social anxiety levels and response inhibition abilities in predicting SB use is further depicted in Fig. 1.

### Moderation of Post-event Processing

The second moderation model tested whether response inhibition abilities (SSRT) moderated the association between social anxiety (high or low) and PEP following a social stressor (SARQ; Table 3). The model was significant (*F*(3,179) = 23.85, *p* < 0.001) and explained 29% of the variance in SARQ scores. The main effect of social anxiety was significant and positive (*b* = 12.35, *SE* = 1.60, 95% CI [9.19, 15.51], *p* < 0.001), and the main effect of response inhibition was not significant (*b* = 0.01, *SE* = 0.01, 95% CI [− 0.02, 0.04], *p* = 0.634). Again, these main effects were qualified by a significant group by SSRT interaction.

**Fig. 2:**
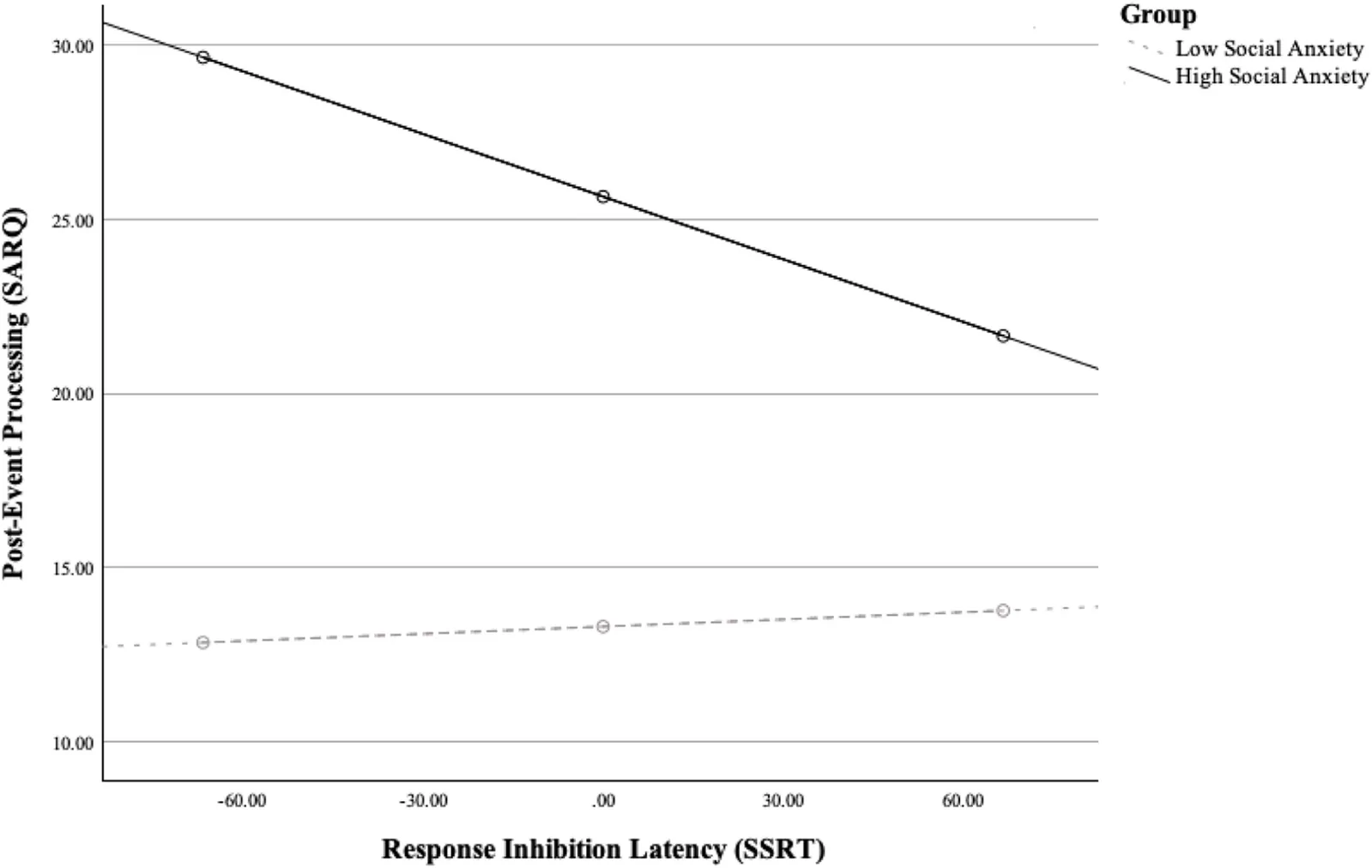
Group differences in post-event processing (Socially Anxious Rumination Questionnaire [SARQ]) by response inhibition ability (Stop Signal Reaction Time [SSRT]; higher values indicate poorer response inhibition)

Indeed, the interaction between social anxiety and response inhibition was significant (*b* = − 0.07, *SE* = 0.03, 95% CI [− 0.12, − 0.02], *p* = 0.011). Conditional effects indicated that group differences in PEP were largest at lower SSRT values (i.e., better inhibition; –1 *SD* SSRT: *b* = 16.81, *SE* = 2.32, *t* = 7.25, *p* < 0.001), and smallest at higher SSRT values (i.e., poorer inhibition; + 1 *SD* SSRT: *b* = 7.89, *SE* = 2.40, *t* = 3.28, *p* = 0.001). The interaction between social anxiety levels and response inhibition abilities in predicting PEP is further depicted in Fig. 2.

## Discussion

The present study examined whether individual differences in response inhibition abilities moderated associations between social anxiety and two core mechanisms involved in the maintenance of SAD: SBs and PEP. Given prior research suggesting that cognitive control plays an important role in facilitating adaptive emotion regulation, coping, and goal-directed behavior (Zelazo, 2020), we predicted that higher response inhibition abilities would correspond to a weaker link between social anxiety and maladaptive avoidance (i.e., SBs) and ruminative (i.e., PEP) behaviors. Consistent with prior research, individuals high in social anxiety reported increased use of both SBs and PEP relative to individuals low in social anxiety (Cuming et al., 2009; Flynn & Yoon, 2025). Response inhibition abilities did not significantly differ between groups or demonstrate significant bivariate correlations with mechanisms of SAD across the full sample. However, response inhibition abilities did interact with social anxiety levels to predict both SBs and PEP, but in an unexpected direction. In fact, the link between social anxiety and maladaptive maintenance behaviors was amplified at *higher* (vs. lower) levels of response inhibition abilities. These findings indicate that response inhibition does not operate uniformly across levels of social anxiety; instead, it might function to either amplify or attenuate anxiety-maintaining behaviors.

One interpretation of the present findings is that response inhibition abilities might be applied differently by individuals with high versus low levels of social anxiety in a manner that is consistent with their divergent goals in social situations. Indeed, anxiety is typically associated with excessive motivation to avoid threats rather than approach rewards (Robin L. & Martin, 2010). SAD in particular is characterized by an over-reliance on avoidance-based emotion regulation strategies (Jazaieri et al., 2014), and an increased salience of such avoidant emotion regulation goals in social contexts (Goodman et al., 2019). Therefore, individuals with social anxiety might be relatively less focused on valued, reward-focused goals in social contexts than non-anxious individuals, who might be relatively more motivated towards personally important, approach-oriented aims such as relationship-building (Goodman et al., 2019). For such non-anxious individuals, their response inhibition skills might be deployed to reduce enactment of avoidance‑oriented behaviors (i.e., SBs) in favor of social approach behaviors, and to facilitate disengagement from repetitive negative thoughts (i.e., PEP) that distract from social rewards (Adamis et al., 2025a, b). This notion is consistent with work linking cognitive control capabilities to decreased repetitive negative thinking and increased adaptive emotion regulation (Fox et al., 2015; Joormann & Gotlib, 2010; Pruessner et al., 2020). However, conclusions drawn from this prior work might be incomplete if individual differences in anxiety are not taken into account.

For individuals with high social anxiety, strong response inhibition abilities might be recruited in service of maladaptive, disorder-consistent goals, such as suppressing and/or avoiding feelings of anxiety (Goodman et al., 2019; Jazaieri et al., 2014), closely monitoring and restricting one’s performance during social events (Clark & Wells, 1995), and striving to meet the vague, yet high perceived social standards of others (Hofmann, 2007). Ultimately, such threat-driven motivations in social contexts function to prevent the core feared outcome in SAD— negative evaluation, embarrassment, or rejection (American Psychiatric Association, 2022). Indeed, for those with high levels of response inhibition coupled with goals focused on conveying a favorable impression of oneself to others (Clark & Wells, 1995; Hofmann, 2007), it follows that one’s cognitive control resources would be deployed towards enacting behaviors that are in line with such goals, such as using SBs to reduce state anxiety and social risks, and engaging in PEP to learn how to avoid future social mishaps. Much like prior research showing that less cognitive control can sometimes be more adaptive (Bocanegra et al., 2014), the present study suggests that more cognitive control is not inherently a protective factor, but rather is conditionally adaptive.

The present findings add to a small but growing literature that indicates that cognitive control might function differently in highly anxious relative to non-anxious populations. For example, Modico et al. (2025) found that attentional control moderated the association between anxiety severity and threat interpretation biases in clinically anxious youth, such that higher attentional shifting abilities *amplified* the relation between anxiety and interpretation biases. Similarly, Ramos et al. (2022) found that attentional shifting abilities *enhanced* the association between anxiety and internal threat/error monitoring (indexed via the error-related negativity [ERN]) in a sample of clinically anxious youth. In an adult sample, it was further found that subjective cognitive control abilities moderated the relation between generalized anxiety and intolerance of uncertainty such that higher perceived attentional control corresponded to a stronger link between intolerance of uncertainty and clinical levels of generalized anxiety and worry (Saulnier et al., 2021). Although at the group level, anxiety tends to be negatively associated with cognitive control abilities on average (Eysenck, 2010; Shi et al., 2019), these results contend with the notion that cognitive control is inherently, universally protective and suggest that it can in fact amplify rather than attenuate maladaptive processes in anxious individuals. Indeed, more contemporary theoretical advancements explaining risk for and development of SAD suggests that “automatic” control abilities (such as the response inhibition latencies assessed in the SST) might interact with behavioral inhibition temperaments to *increase* risk for social anxiety by promoting maladaptive attentional deployment in social contexts, whereas “planful” control abilities (e.g., task switching, proactive control) might decrease anxiety vulnerability by promoting more adaptive information processing (Fox et al., 2021; Henderson & Wilson, 2017; Henderson et al., 2015). Taken together, both theoretical and empirical work suggests that amongst individuals with high levels of anxiety and/or anxiety vulnerability, cognitive control resources might be applied towards focusing on threatening interpretations of ambiguous scenarios (Modico et al., 2025), closely monitoring one’s performance and errors (Ramos et al., 2022), engaging in worry to reduce uncertainty (Saulnier et al., 2021), and enacting restricted and harshly evaluated social behaviors to the extent that one is principally motivated to avoid threats (Robin L. & Martin, 2010).

Results from the present study add nuance to our understanding of the role of cognitive control in anxiety. More specifically, these findings suggest that cognitive control might not be ubiquitously adaptive, and instead can be applied towards the enactment of threat-avoidant behaviors that maintain social anxiety (Modico et al., 2025; Ramos et al., 2022; Saulnier et al., 2021). There are some potential clinical implications of these results that might inform enhanced treatments for SAD. Most importantly, the present results suggest that a mindful understanding and approach towards leveraging cognitive control abilities is necessary in the treatment of social anxiety. Indeed, cognitive control might only be protective insofar as it is applied towards adaptive rather than threat-driven, avoidant goals. Thus, psychoeducation on the (dys)functional role of patients’ engagement in anxiety-maintaining behaviors such as SBs and PEP is paramount (Clark & Wells, 1995). Indeed, in the absence of collaborating with patients to conduct a functional analysis that explores the detrimental long-term effects of SBs and PEP, patients with strong cognitive control might have difficulty reducing such behaviors, not because the patient is unable to inhibit them, but rather because they are aligned with maladaptive short-term goals (e.g., avoidance of anxiety, impression management). Future research is needed to examine if specific intervention strategies for reducing SB use (e.g., behavioral experiments, text message reminders; Hofmann, 2007; Zech et al., 2025) and PEP (e.g., distraction, mindfulness; Hilt & Pollak, 2012) might be augmented by therapies targeting cognitive control (e.g., du Toit et al., 2020) once patients with social anxiety have set and “bought in” to goals related to inhibiting these anxiety-maintaining behaviors.

There are several limitations of the present study that highlight important areas for future research. First, we employed a case–control design and recruited an analogue sample of individuals endorsing high levels of social anxiety symptoms. Thus, future research is needed to replicate the present findings in a sample representing the full spectrum of social anxiety severity, including patients meeting clinical criteria for SAD, to enhance the generalizability of results. Second, while we assessed response inhibition abilities using a well-validated, objective behavioral paradigm (the SST), SB use and PEP were measured via self-report questionnaires, which are subject to important response biases. Future research might incorporate behavioral coding and/or experience sampling methods to improve the reliability and validity of SB and PEP assessments. Relatedly, future research would also benefit from incorporating a measure of proactive (vs. reactive) inhibitory control, which might play a different functional role in moderating the association between social anxiety and use of SBs/PEP. Third, although the impromptu speech task offered a standardized social stressor around which to anchor PEP assessments, such laboratory-based tasks might not fully replicate the salience and interpersonal complexity of real-world social stressors. Future research is needed to examine the conditional effects of response inhibition on PEP surrounding more realistic, day-to-day social scenarios. Fourth, although theory and prior empirical work imply that individuals with social anxiety differ from non-anxious individuals in terms of their regulatory and social goals (Clark & Wells, 1995; Goodman et al., 2019; Hofmann, 2007), we did not assess for participants’ motivations surrounding their SB use and PEP. Such data will be important for further understanding the potential functional role that response inhibition plays in facilitating goal-directed behavior to either enhance or attenuate the maintenance of social anxiety. Relatedly, there are several potential confounds that were not measured in the present study that might explain the observed patterns of associations. For example, group differences in trait impulsivity (Lipton et al., 2016), error monitoring (Moser et al., 2013), comorbidities involving executive functioning impairments (e.g., attention-deficit hyperactivity disorder; Jakobsson Store et al., 2024), or other individual differences associated with both social anxiety and response inhibition might explain meaningful variance in the observed interaction between social anxiety and SSRT in predicting SBs and PEP. This represents an important area to investigate further in future research. Despite these limitations, the present findings highlight the importance of carefully considering individual differences in cognitive control when conceptualizing factors underlying the maintenance of social anxiety. Understanding when and why response inhibition shifts from protective to maladaptive in the context of SAD and other anxiety disorders offers a compelling direction for future research that might help refine cognitive-behavioral interventions.

## Supplementary Information

Below is the link to the electronic supplementary material.

Supplementary Material 1

## Data Availability

Data will be made available upon request.
