# Supplementary material for: Does Cognitive Control Help or Hurt Social Anxiety? Conditional Effects of Response Inhibition on Safety Behavior Use and Post-event Processing
Source: Cognit Ther Res. 2026 Apr 24;50(5):1035–45. doi: 10.1007/s10608-026-10746-x (PMC13166126; doi:10.1007/s10608-026-10746-x)
Supplement: Supplementary file 1 [file 10608_2026_10746_MOESM1_ESM.docx]

*Supplemental Figure 1.* Q-Q plot of the standardized residuals of Model 1 (testing the interaction of social anxiety and response inhibition predicting safety behavior use)

*Supplemental Figure 2.* Q-Q plot of the standardized residuals of Model 2 (testing the interaction of social anxiety and response inhibition predicting post-event processing)

Supplemental Table 1. Unstandardized Model Coefficients for the Interaction of Social Anxiety Severity and Response Inhibition Predicting Safety Behaviors and Post‑Event Processing

| Predictor | Y_1_ Safety Behaviors  (*n* = 192) | | | Y_2_ Post-Event Processing  (*n* = 183) | | |
| --- | --- | --- | --- | --- | --- | --- |
|  | *b* | *SE* | *p* | *b* | *SE* | *p* |
| SIAS | 0.76 | 0.07 | .00 | 0.58 | 0.67 | .00 |
| Response Inhibition | 0.01 | 0.02 | .47 | -0.02 | 0.01 | .06 |
| SIAS*Response Inhibition | 0.00 | 0.00 | .80 | 0.00 | 0.00 | .67 |
| Constant | 31.01 | 1.05 | .00 | 20.17 | 0.67 | .00 |
|  | R^2^ = .39 | | | R^2^ = .49 | | |
|  | *F*(3, 188) = 39.43 | | | *F*(3, 179) = 56.83 | | |

*Note*. SIAS = Social Interaction Anxiety Scale; Response Inhibition = Stop Signal Reaction Time (higher values indicate poorer inhibitory control); Safety Behaviors = Subtle Avoidance Frequency Estimation scores; Post-Event Processing = Socially Anxious Rumination Questionnaire scores.
